# Development and verification of a lipotoxicity-related gene signature for predicting prognosis and immune landscape in colorectal cancer

**DOI:** 10.1007/s12672-026-05206-7

**Published:** 2026-05-20

**Authors:** Chenlu Xiong, Pinglian Liao, Jin Li

**Affiliations:** Department of Anesthesiology, Gaoxin Branch of The First Affiliated Hospital, Jiangxi Medical College, Nanchang University, Nanchang, 330096 China

**Keywords:** Colorectal cancer, Lipotoxicity, Immune microenvironment, Prognostic model

## Abstract

**Background:**

Colorectal cancer (CRC), a lethal tumor of the intestine, has lipotoxicity, which exerts a significant influence on the onset and development of intestinal disorders. This study provides an in-depth evaluation of lipotoxicity-related genes (LRGs) in the CRC treatment setting.

**Methods:**

Differentially expressed genes (DEGs) within The Cancer Genome Atlas-CRC dataset and LRGs were intersected to obtain the LRG-DEGs. Subsequently, univariate Cox regression and a random survival forest model were conducted to select prognostic LRGs and construct a risk model for patients with CRC. Then, prognosis was further investigated via independent prognostic analysis, nomogram construction, enrichment analysis, immune microenvironment analysis, and drug sensitivity testing. Finally, reverse transcription quantitative PCR (RT-qPCR) was used to verify the expression levels of prognostic LRGs.

**Results:**

The constructed risk model with five prognostic LRGs (*PPARGC1A*, *CPT2*, *CXCL1*, *FABP4*, and *OFCC1*) showed high prognostic effectiveness. The nomogram subsequently built based on risk score, age, T stage, and M stage showed strong prognostic power. Moreover, the enrichment of diverse pathways was observed across distinct risk groups, exemplified by the PPAR signaling pathway and complement and coagulation cascades. Analysis of the immune microenvironment revealed the strongest positive association between *FABP4* and natural killer cells. Drug sensitivity testing identified efficacious drugs for patients with CRC, such as midostaurin and lenalidomide. Notably, RT-qPCR confirmed elevated expression levels for *CXCL1* and *OFCC1* and reduced levels for *FABP4*, *PPARGC1A*, and *CPT2* in patients with CRC.

**Conclusion:**

Five prognostic LRGs were determined for CRC, and a new risk model was developed and validated, revealing the critical role of LRGs in CRC and improving our understanding of clinical interventions for this cancer type.

## Introduction

Colorectal cancer (CRC), the third most diagnosed malignancy and second leading cause of cancer-related mortality globally, arises through heterogeneous pathways, including adenoma-carcinoma progression, serrated lesions, and inflammatory mechanisms [1]. Consensus molecular subtype classification categorizes CRC types based on genomic and transcriptomic profiles. Despite advances in surgical resection, chemotherapy, and targeted therapies (e.g., anti-epidermal growth factor receptor/vascular endothelial growth factor [VEGF] agents), 5-year survival remains suboptimal (65%–70%) due to high recurrence rates (30%–40% in advanced stages) and therapeutic resistance [2–4]. Although precision medicine and bioinformatics have enhanced multi-gene targeting strategies, surpassing the efficacy of single-gene approaches, current therapies face a plateau in improving health outcomes [3, 5]. Therefore, there is an urgent need for novel therapeutic paradigms to address unmet clinical challenges and refine prognostic prediction, particularly through immune microenvironment modulation and the exploration of lipotoxicity-related pathways.

Lipotoxicity, a phenomenon characterized by ectopic lipid accumulation in non-adipose tissues, disrupts cellular homeostasis through inflammation, oxidative stress, and mitochondrial dysfunction, thereby contributing to metabolic disorders such as diabetes as well as to organ damage. Emerging evidence also suggests its potential role in CRC [6]. High-fat diets induce intestinal lipotoxicity by increasing luminal free fatty acids (FFAs), which impair gut immunity via cytotoxicity to intestinal T cells, reducing intraepithelial and lamina propria lymphocytes [7, 8]. Sustained depletion of these immune cells exacerbates intestinal injury and may promote CRC pathogenesis. In Japan, westernized diets linked to increased consumption of high-fat foods correlate with rising CRC incidence, potentially mediated by FFA-driven mucosal damage and immune dysfunction [7]. However, the precise mechanisms through which lipotoxicity-related genes (LRGs) influence CRC biology remain unexplored. Elucidating the roles of LRGs in CRC could unveil novel biomarkers or therapeutic targets to mitigate diet-associated oncogenesis [9].

To address this knowledge gap, in this study, CRC-related datasets obtained from the Gene Expression Omnibus (GEO) and The Cancer Genome Atlas (TCGA) databases were screened for prognostic LRGs in CRC, and a novel CRC risk model was built. Additionally, we investigated the roles of these prognostic genes in the tumor immune microenvironment (as well as their pathways and functions) and established molecular networks based on them. Finally, we explored the sensitivity of patients at different risk levels to common chemotherapeutic agents. These findings aim to provide novel insights and strategies for the diagnosis and treatment of CRC.

## Materials and methods

### Data acquisition

The RNA-sequencing (RNA-seq) profiles and associated clinical records of patients with CRC were obtained from the UCSC Xena database (https://xena.ucsc.edu/) on October 10, 2024. The training datasets, namely TCGA-READ and TCGA-COAD (collectively referred to as TCGA-CRC), comprised 606 CRC samples and 51 control samples. The GSE87211 RNA-seq datasets relevant to CRC were obtained from the GEO database (http://www.ncbi.nlm.nih.gov/geo/). Among them, 363 CRC samples were retained in the validation set GSE87211 (GPL13497 platform). At the same time, 196 patients with CRC of GSE87211 with complete survival information and gene expression were analyzed to validate the prognostic model.

In addition, 467 LRGs were retrieved from the GeneCard database (https://www.genecards.org/) [10].

### Differential expression analysis

To detect differentially expressed genes (DEGs) related to CRC, the TCGA-CRC dataset (control and CRC samples) was subjected to differential expression analysis using the DESeq2 package (v 1.38.0) [11] with adj.*p* < 0.05 and |log_2_fold change (FC)| > 1.0. The top five upregulated/downregulated DEGs sorted in descending order based on log_2_ FC values were visualized via volcano plots and heat maps generated using the ggplot2 package (v 3.4.4) [12] and ComplexHeatmap package (v 2.14.0) [13].

### Recognition and functional enrichment analysis of LRG-DEGs

LRG-DEGs were determined based on the overlap between DEGs and LRGs. Subsequently, Gene Ontology (GO) and Kyoto Encyclopedia of Genes and Genomes (KEGG) enrichment analyses (*p* < 0.05) were conducted to elucidate the biological functions and signaling pathways of LRG-DEGs involved in CRC development using the ClusterProfiler package (v 4.7.1.003) [14]. Next, LRG-DEGs were subjected to protein–protein interaction (PPI) analysis using the STRING database (http://www.string-db.org/) (interaction score > 0.15) and Cytoscape software (v 3.9.1) [15].

### Screening of prognostic LRGs and risk model construction

LRG-DEGs within the TCGA-CRC dataset for patients with CRC were subjected to proportional hazards (PHs) assumption testing (*p* > 0.05) and univariate Cox regression analysis (hazard ratio [HR] ≠ 1, *p* < 0.01) using the survival package (v 3.5.3) [16] and survminer package (v 0.4.9) [17], respectively, to obtain prognostic LRGs. Subsequently, a random survival forest (RSF) model was applied to them using the randomForestSRC package (v 3.2.2) [18]. Using these prognostic LRGs, the risk scores for each patient were computed by applying the following equation:

$$h(t|{\mathrm{x}})=\frac{1}{B}\,\sum\limits_{{t=1}}^{B} {{h_l}} (t|{\mathrm{x}}),$$

where *h*_*i*_(*t*|) is the prediction result of the *i*th tree for an individual at time *t*. Next, patients with CRC from the TCGA-CRC and GSE87211 datasets were categorized into two risk groups (high and low) based on median risk scores. A risk curve plot, survival status plot, and heat map were generated to visualize the distributions of CRC samples and the expression levels of prognostic LRGs. The survminer package (v 0.4.9) [17] and survivalROC package (v 1.0.3.1) were used to generate Kaplan–Meier (KM) curves and receiver operating characteristic (ROC) curves to assess the risk model’s prognostic effectiveness, respectively. Area under the curve (AUC) values for overall survival (OS) periods of 1, 2, and 3 years > 0.6 indicated that the model had some discriminatory power for patients with CRC.

### Establishment and appraisal of a predictive nomogram

To assess the significance of the risk model’s impact on OS in patients with CRC and obtain independent prognostic factors, clinical features in the TCGA-CRC dataset were subjected to PH assumption testing (*p* > 0.05) and both univariate and multivariate Cox regression analyses (*p* < 0.05) using the survminer package (v 0.4.9).

Subsequently, a nomogram was established using the rms package (v 6.5.0) [19] for the prognosis of 1-, 2-, and 3-year OS in patients with CRC. ROC and calibration curves were applied to estimate the nomogram’s predictive effectiveness using the survivalROC package (v 1.0.3.1) and rms package (v 6.5.0), respectively.

### Gene set enrichment analysis (GSEA) and gene–gene interaction (GGI) network

The TCGA-CRC dataset was subjected to GSEA in clusterProfiler (v 4.7.1.003) to determine the disparities in pathways between the high- and low-risk groups. Differential analysis of the two groups was performed using the DESeq2 package (v 1.38.0), and the calculated log_2_FC values were ranked from large to small to obtain the corresponding list of related genes. The “c2.cp.kegg.v7.4.symbols.gmt” acquired from the Molecular Signatures Database (MsigDB; https://www.gsea-msigdb.org/gsea/msigdb) was used as the background set for GSEA (*p* < 0.05).

Furthermore, a GGI network was generated for the prognostic LRGs on the GeneMANIA website (http://www.genemania.org/), showcasing the top 20 genes associated with these prognostic LRGs and related functions.

### Immune microenvironment analysis

Understanding immune cell infiltration is critical for elucidating disease defense mechanisms, and determining its magnitude can offer valuable insights into CRC pathogenesis. Initially, the ssGSEA algorithm in the GSVA package (v 1.46.0) [20] was adopted to determine enrichment scores for 28 types of infiltrating immune cells [21] between the two risk groups (high and low) in the TCGA-CRC dataset, which were then analyzed via the Wilcoxon test (*p* < 0.05) using the rstatix package (v 0.7.2) [22]. Moreover, Spearman correlation analysis was conducted to illustrate the associations between differential immune cells and prognostic LRGs using the psych package (v 2.2.9) [23] (|correlation coefficient (cor)| > 0.30 and *p* < 0.05).

Moreover, the expression of 48 immune checkpoints was analyzed between high- and low-risk groups via the Wilcoxon test (*p* < 0.05) using the rstatix package (v 0.7.2) to explore potential novel immunotherapy targets [24]. The disparities in immune and stromal cell content in the immune microenvironment were elucidated by computing the immune score, stromal score, and ESTIMATE score for the two risk groups using the ESTIMATE algorithm in the estimate package (v 1.0.13) [25] and the Wilcoxon test (*p* < 0.05). To evaluate differences in the responses of high- and low-risk patients to immunotherapy, we examined the correlation between the risk score and tumor immune dysfunction and exclusion (TIDE) score (|cor| > 0.30 and *p* < 0.05) and then applied the Wilcoxon test to compare differences in TIDE scores between high- and low-risk patients with CRC (*p* < 0.05).

### Genomic variation analysis and drug sensitivity testing

Somatic mutation data within the TCGA-CRC dataset were analyzed using the maftools package (v 2.14.0) [26] to assess differences in genomic mutations between the two risk groups. At the same time, waterfall plots were generated to separately illustrate the distribution of the 20 most frequently mutated genes in both groups, and an additional waterfall plot was created to display the mutation types in prognostic LRGs.

Drug sensitivity analysis was carried out using the GDSC database (https://www.cancerrxgene.org/) to provide recommendations for CRC management. The half-maximal inhibitory concentration (IC_50_) of common chemotherapeutic and molecular-targeted agents corresponding to each patient with CRC in the TCGA-CRC dataset was calculated using the pRRophetic package (v 0.5) [27] to infer drug sensitivity. Differences in the sensitivity to medications commonly used in the clinical treatment of CRC between high- and low-risk groups were comparatively analyzed using the Wilcoxon test (*p* < 0.05).

### Molecular regulatory network construction and expression analysis related to prognostic LRGs

The microRNAs (miRNAs) of prognostic LRGs were predicted based on the miRanda and miRDB databases using the multiMiR package (v 1.20.0) [28]. Subsequently, the upstream long non-coding RNAs (lncRNAs) of these common miRNAs were predicted based on the StarBase database (https://link.zhihu.com/?target=http%3 A//starbase.sysu.edu.cn/). The NetworkAnalyst database (https://www.networkanalyst.ca/) with JASPAR as the background was utilized to search for transcription factors (TFs) that regulate the prognostic LRGs. The lncRNA–miRNA–mRNA and mRNA–TF regulatory networks were visualized using the ggraph package (v 2.1.0) [29] and Cytoscape software (v 3.9.1).

Additionally, the expression profiles of prognostic LRGs in the TCGA-CRC dataset were investigated by comparing gene expression levels between the control and CRC groups using the Wilcoxon test (*p* < 0.05).

### Experimental validation by reverse transcription quantitative PCR (RT-qPCR)

Tissue specimens of five patients with CRC and five control individuals were collected at the Gaoxin Branch of the First Affiliated Hospital, Jiangxi Medical College, Nanchang University. All participants filled out an informed consent form before tissue collection. The ethical authorization (approval number [2023] No.19) was granted by the Ethics Committee of Gaoxin Branch of The First Affiliated Hospital, Jiangxi Medical College, Nanchang University. All methods were carried out in accordance with relevant guidelines and regulations. Informed consent was obtained from all subjects. Total RNA was separated with Trizol reagent (Ambion, Texas, USA) and then used to obtain cDNA. PCR amplification was carried out in a 10-µL reaction mixture consisting of 3 µL of cDNA, 5 µL of 2x UnivYBR Green qPCR Master Mix, and 1 µL of each forward and reverse primer. The thermal cycling parameters were set as follows: a pre-denaturation step at 95 °C for 1 min, 40 cycles of denaturation at 95 °C for 20 s, annealing at 55 °C for 20 s, and extension at 72 °C for 30 s. The relative mRNA expression levels of prognostic LRGs were measured using the 2^−ΔΔCt^ method. The RT-qPCR primer pairs (sequences included in Table 1) were designed by Sangon Biotech (Shanghai, China). *GAPDH* was used as the internal reference gene to guarantee accurate normalization.

### Statistical analysis

All data were analyzed using R language software (v 4.2.2) and GraphPad Prism (v 10). The t-test was adopted to compare the relative mRNA expression levels of prognostic LRGs. Comparisons were analyzed using the Wilcoxon test, with statistical significance established at p-value < 0.05.

## Results

### Recognition and functional evaluation of 118 LRG-DEGs

**Fig. 1:**
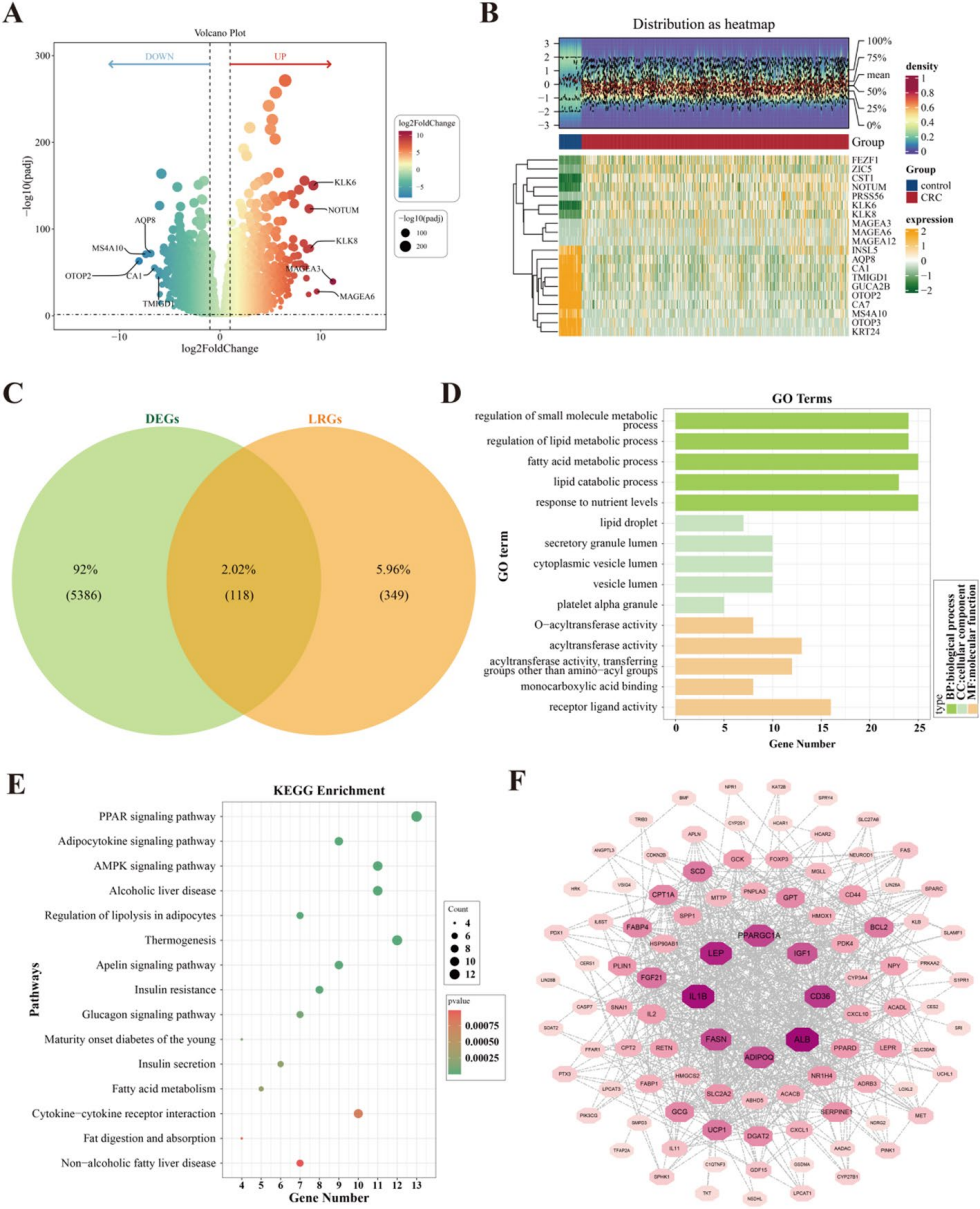
Identification and functional analysis of lipotoxicity-related differentially expressed genes (LRG-DEGs) in colorectal cancer (CRC). **A** Volcano plot displaying 5,504 differentially expressed genes (DEGs) between CRC and control samples (|log₂FC| >1.0, adj.*p* < 0.05), with red and blue dots indicating significantly upregulated and downregulated genes, respectively. **B** Heatmap of the top 5 upregulated/downregulated DEGs in CRC samples. **C** Venn diagram intersecting 5,504 DEGs with 467 lipotoxicity-related genes (LRGs), yielding 118 LRG-DEGs. **D** GO enrichment analysis showing top terms (e.g., regulation of lipid metabolic processes, lipid droplet formation). **E** KEGG pathway enrichment (e.g., PPAR signaling and adipocytokine pathway). **F** Protein–protein interaction (PPI) network for LRG-DEGs highlighting hub genes (*PPARGC1A*, *LEP*, and *IL1B*). (All analyses are based on the TCGA-CRC cohort; *p* < 0.05)

Differential expression analysis of the TCGA-CRC dataset for the CRC group yielded a total of 5,504 DEGs, 2,859 upregulated and 2,645 downregulated. The markings and expression levels of these DEGs were visualized via a volcano plot and heat map (Fig. 1A, B). The overlapping of 5,504 DEGs and 467 LRGs revealed a total of 118 LRG-DEGs (Fig. 1C). These were then subjected to GO and KEGG enrichment analyses to elucidate molecular biological processes. Notably, LRG-DEGs were significantly enriched in 1,357 GO terms, including regulation of lipid metabolic processes, lipid droplet, O-acyltransferase activity, and acyltransferase activity (Fig. 1D). KEGG analysis revealed enrichment in 52 functional pathways, including PPAR signaling, adipocytokine signaling, alcoholic liver disease, and AMPK signaling (Fig. 1E). Subsequent analysis of protein interactions with LRG-DEGs revealed a protein interaction network comprising 99 proteins and 697 interaction pairs. Within this network, *PPARGC1A*, *LEP*, *IL1B*, *FASN*, *ADIPOQ*, *ALB*, *CD36*, and *IGF1* genes exhibited extensive interactions with other genes (Fig. 1F). These analyses highlight promising pathways of CRC linking LRG-DEGs for therapeutic targeting.

### Development and verification of the prognostic model for CRC

**Fig. 2:**
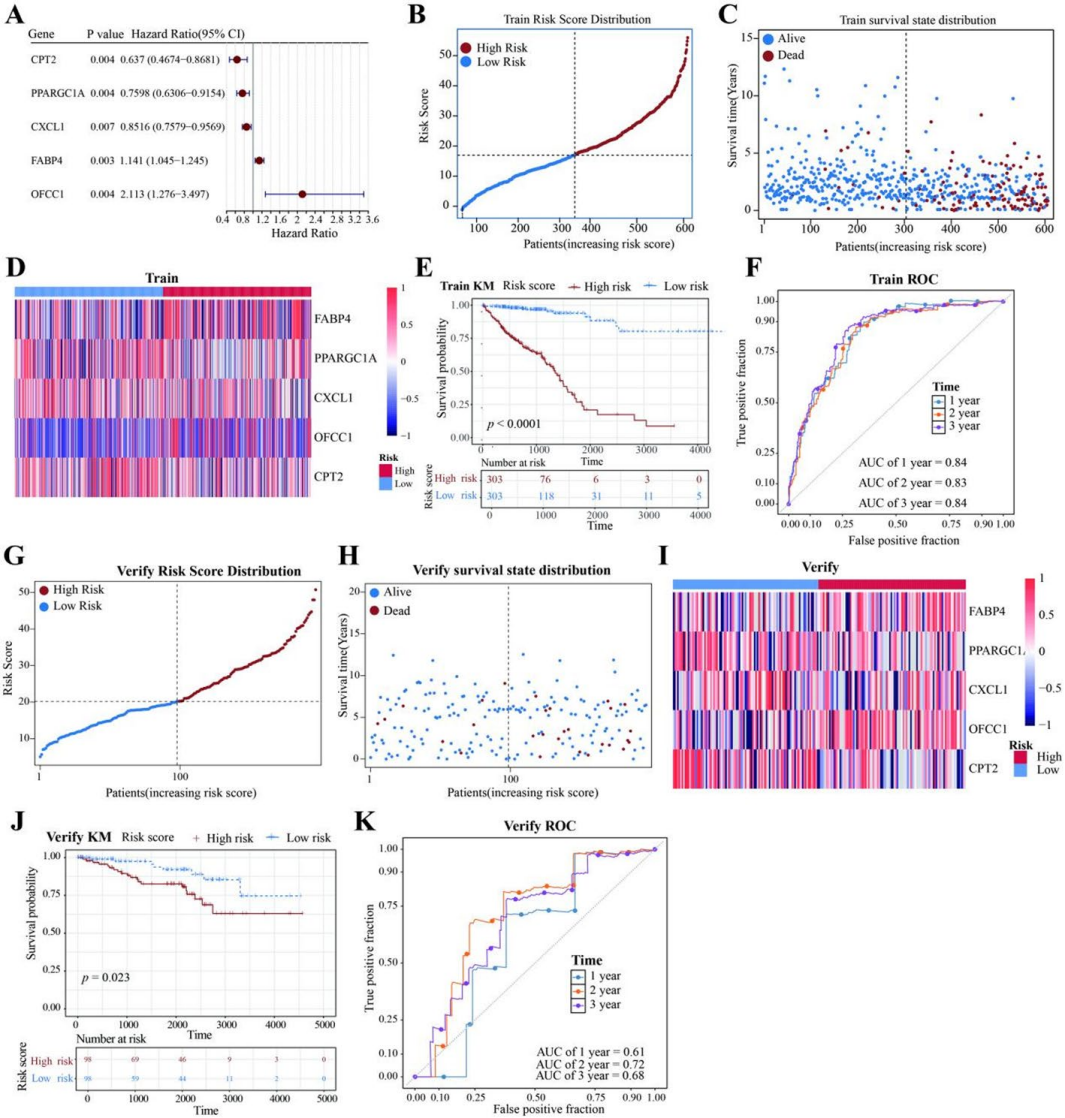
Construction and validation of the prognostic risk model based on five LRGs. **A** Univariate Cox regression identifying five prognostic LRGs: *PPARGC1A*, *CPT2*, *CXCL1*, *FABP4*, and *OFCC1*. The first three were identified as protective factors, whereas the remaining two were identified as risk factors. **B–D** Risk score distribution, survival status, and gene expression heatmap for the TCGA training dataset. The high-risk group showed increased mortality and altered gene expression. **E** KM curves confirming poorer overall survival (OS) in high-risk patients (*p* < 0.0001). **F** Time-dependent ROC curves (AUC 1/2/3-year: 0.84/0.83/0.84). **G–L** Consistent validation in the GSE87211 cohort (risk distribution, survival status, KM, and ROC). High/low-risk groups are stratified based on the median risk score (18.24)

The univariate Cox regression analysis and PH assumption test identified five prognostic LRGs significantly with and OS for the CRC group in the TCGA-CRC dataset. Specifically, *PPARGC1A*, *CPT2*, and *CXCL1* were identified as protective factors against CRC (HR < 1), whereas *FABP4* and *OFCC1* were recognized as risk factors (HR > 1) (Fig. 2A). These five prognostic LRGs (*PPARGC1A*, *CPT2*, *CXCL1*, *FABP4*, and *OFCC1*) were used to establish an RSF model in order to enhance gene selection and obtain a more reliable risk model. Moreover, the CRC samples in the TCGA-CRC dataset were differentiated into two risk groups (high/low) using a median risk score of 18.24376 (high-/low-risk patients = 303/303). The risk profile and survival condition plot in Fig. 2B, C show that, in the high-risk group, mortality increased as the risk score rose, and the expression levels of *PPARGC1A*, *CPT2*, and *CXCL1* were lower, whereas those of *FABP4* and *OFCC1* were higher (Fig. 2D). Based on the KM curves, the high-risk group exhibited reduced OS duration and a bleak prognosis (*p* < 0.0001) (Fig. 2E). The AUC values confirmed the strong predictive power of the prognostic model in estimating OS for patients with CRC (AUC = 0.84, 0.83, and 0.84 at 1, 2, and 3 years, respectively; Fig. 2F). Moreover, the prognostic model’s stability was tested using the GSE87211 dataset, yielding results comparable to those obtained using the TCGA-CRC dataset (Fig. 2G–K). These findings further proved the universality of the risk models.

### Nomogram for CRC based on independent risk factors

**Fig. 3:**
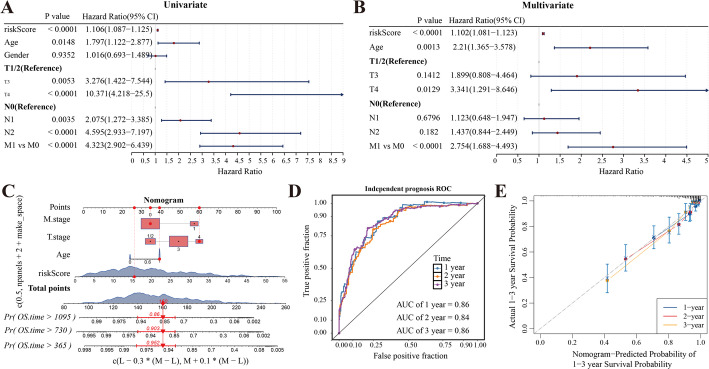
Nomogram integrating independent prognostic factors. **A**–**B** Forest plots from univariate (**A**) and multivariate (**B**) Cox regression analyses confirming independent prognostic values for risk score, age, T4 stage, and M stage (all *p* < 0.05). **C** Nomogram-based prediction of 1-, 2-, and 3-year overall survival (OS) using risk score and clinical factors. **D** ROC curves validating nomogram accuracy (AUC 1/2/3-year: 0.86/0.84/0.86). **E** Calibration curves showing agreement between predicted and observed survival. (Analyses are based on the TCGA-CRC cohort.)

Independent prognostic analyses are essential for establishing robust clinical decision support systems. Hence, within the TCGA-CRC dataset, risk score (HR = 1.102, *p* < 0.05, 95% confidence interval [CI] = 1.081–1.123), age (HR = 2.210, *p* < 0.05, 95% CI = 1.365–3.578), T4 stage (HR = 3.341, *p* < 0.05, 95% CI = 1.291–8.646), and M stage (HR = 2.754, *p* < 0.05, 95% CI = 1.688–4.493) were regarded as independent risk factors based on the results of PH assumption tests (*p* > 0.05) and both univariate and multivariate Cox regression analyses (Fig. 3A, B).

A nomogram was established using risk score, age, T stage, and M stage to demonstrate the predictive accuracy and clinical utility of patients with CRC at 1, 2, and 3 years (Fig. 3C). The ROC curves demonstrated the nomogram’s accurate predictive capabilities, and AUC values were 0.86, 0.84, and 0.86 for OS durations of 1, 2, and 3 years, respectively (Fig. 3D). Then calibration curves demonstrated a good fit between the reference line and the predicted probability of survival in the nomogram at 1, 2, and 3 years (Fig. 3E). These independent risk factors might provide some guidance for more personalized and effective strategies to treat CRC associated with lipotoxicity.

### Biological pathways in CRC

**Fig. 4:**
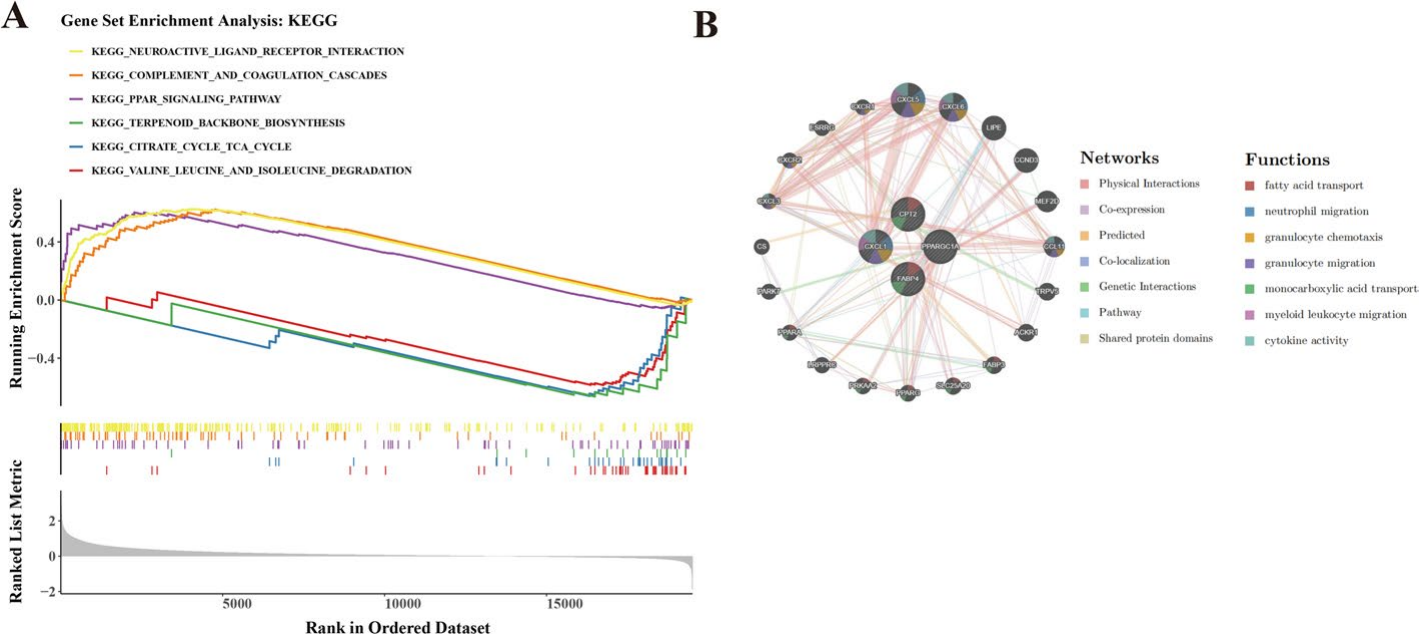
Analysis of pathway enrichment and gene interactions. **A** Gene set enrichment analysis (GSEA) revealing enriched pathways in the high-risk group (e.g., TCA cycle, PPAR signaling, complement cascades; *p* < 0.05). **B** Gene–gene interaction (GGI) network of prognostic LRGs including the top 20 associated genes (e.g., *CXCL5* and *PPARG*) linked to functions like fatty acid transport and neutrophil migration. (GSEA used KEGG gene sets; interactions obtained using GeneMANIA.)

GSEA is particularly valuable for thoroughly elucidating variations in biological processes. Comparative analysis of the high- and low-risk groups within the TCGA-CRC dataset via GSEA revealed 24 significant pathways, including the citrate cycle, tricarboxylic acid (TCA) cycle, terpenoid backbone biosynthesis, PPAR signaling, and complement and coagulation cascades (Fig. 4A). The GGI network revealed associations between prognostic LRGs and several other genes, including *CXCL5*, *CXCL6*, *LIPE*, *CCND3*, *MEF2D*, *CCL11*, *TRPV5*, *ACKR1*, *FABP3*, *SLC25A20*, *PPARG*, *PRKAA2*, *LRPPRC*, *PPARA*, *PARK7*, *CS*, *CXCL3*, *CXCR2*, *ESRRG*, and *CXCR1*, as well as connections to functions like fatty acid transport, neutrophil migration, granulocyte chemotaxis, granulocyte migration, and monocarboxylic acid transport (Fig. 4B). These findings contribute to deepening our understanding of the biological roles associated with lipotoxicity in CRC, offering valuable insights into CRC pathogenesis.

### Tumor microenvironment analysis based on risk scores

**Fig. 5:**
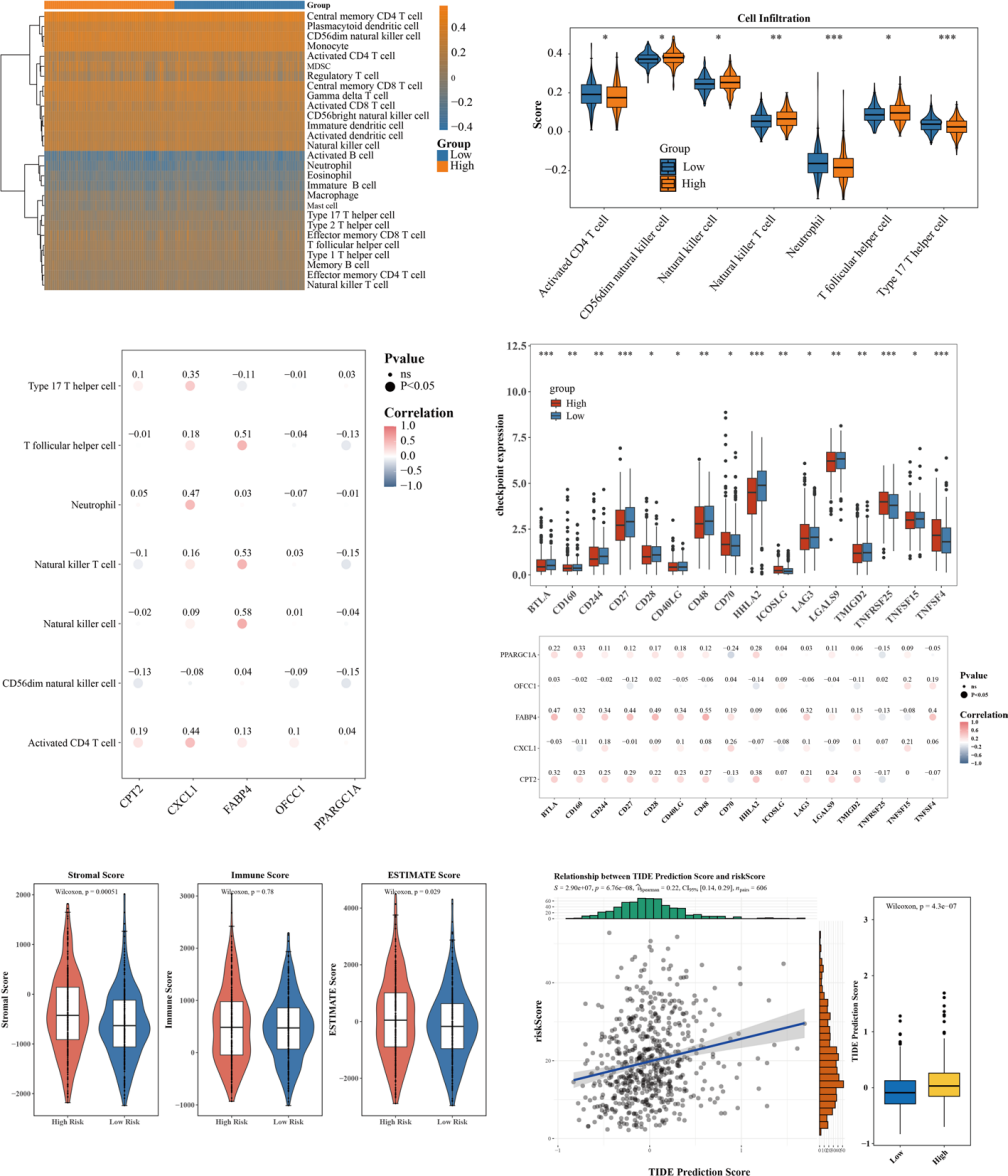
Tumor immune microenvironment analysis. **A** Heatmap of infiltration scores for 28 immune cell types. **B** Boxplot showing differential infiltration: the high- and low-risk groups were enriched in natural killer (NK) subsets and Th17 cells/neutrophils, respectively (*p* < 0.05). **C** Correlation heatmap highlighting FABP4-NK cell association (cor = 0.58, *p* < 0.05). **D** Differential expression of 16 immune checkpoints (e.g., LAG3 and CD48) between risk groups. **E** Strong positive correlation between FABP4 and CD48 (cor = 0.55, *p* < 0.05). **F** Higher stromal/ESTIMATE scores detected in the high-risk group (*p* < 0.05). **G** Elevated TIDE scores in high-risk patients (*p* < 0.05), indicating immune evasion. (All analyses are based on the TCGA-CRC cohort)

Microenvironmental cells are integral components of tissue, and a growing body of evidence shows their clinical and pathological significance in predicting prognosis and therapeutic outcomes [30]. Comparison of immune cell infiltration scores between high- and low-risk groups within the TCGA-CRC dataset demonstrated marked dissimilarities in seven differential immune cell types. Notably, CD56dim natural killer (NK) cells, NK cells, NK T cells, and T follicular helper cells showed higher levels of infiltration in the high-risk group, whereas type 17 T helper cells, neutrophils, and activated CD4 T cells showed higher infiltration in the low-risk group (*p* < 0.05) (Fig. 5A, B). As shown in the correlation heatmap in Fig. 5C, the strongest positive correlation was detected between *FABP4* and NK cells (cor = 0.58, *p* < 0.05), followed by a distinct positive relationship between *FABP4* and NK T cells (cor = 0.53, *p* < 0.05).

To further compare immunological effects between high- and low-risk groups, the expression of immune checkpoint genes was incorporated into differential analysis, revealing significant differences in 16 of them (i.e., *BTLA*, *CD160*, *CD244*, *CD27*, *CD28*, *CD40LG*, *CD48*, *CD70*, *HHLA2*, *ICOSLG*, *LAG3*, *LGALS9*, *TMIGD2*, *TNFRSF25*, *TNFSF15*, and *TNFSF4*) (*p* < 0.05) (Fig. 5D). The strongest positive correlation was detected between CD48 and *FABP4* (cor = 0.55, *p* < 0.05) (Fig. 5E).

Upon further examination of immune infiltration in CRC samples, it was noted that the higher stromal score and ESTIMATE score in the high-risk group might imply that stromal cells in this patient group could be more active in the tumor microenvironment (*p* < 0.05), participating in the growth, invasion, and metastasis of CRC tumors (Fig. 5F). Comparative analysis of TIDE, another immune response indicator, in the high- and low-risk groups revealed higher scores (*p* < 0.05) in high-risk patients. This group also exhibited a certain positive association with the risk score (cor > 0.3, *p* < 0.05), suggesting a stronger propensity for immune escape, which led to a poor prognostic outcome (Fig. 5G). These findings indicated that abnormal immune infiltration, differential expression of immune checkpoints, and immunotherapy response could serve as targets and prognostic markers for CRC associated with lipotoxicity, as they possess crucial immunological and clinical significance.

### Somatic mutations and common antitumor drugs under different risk scores

**Fig. 6:**
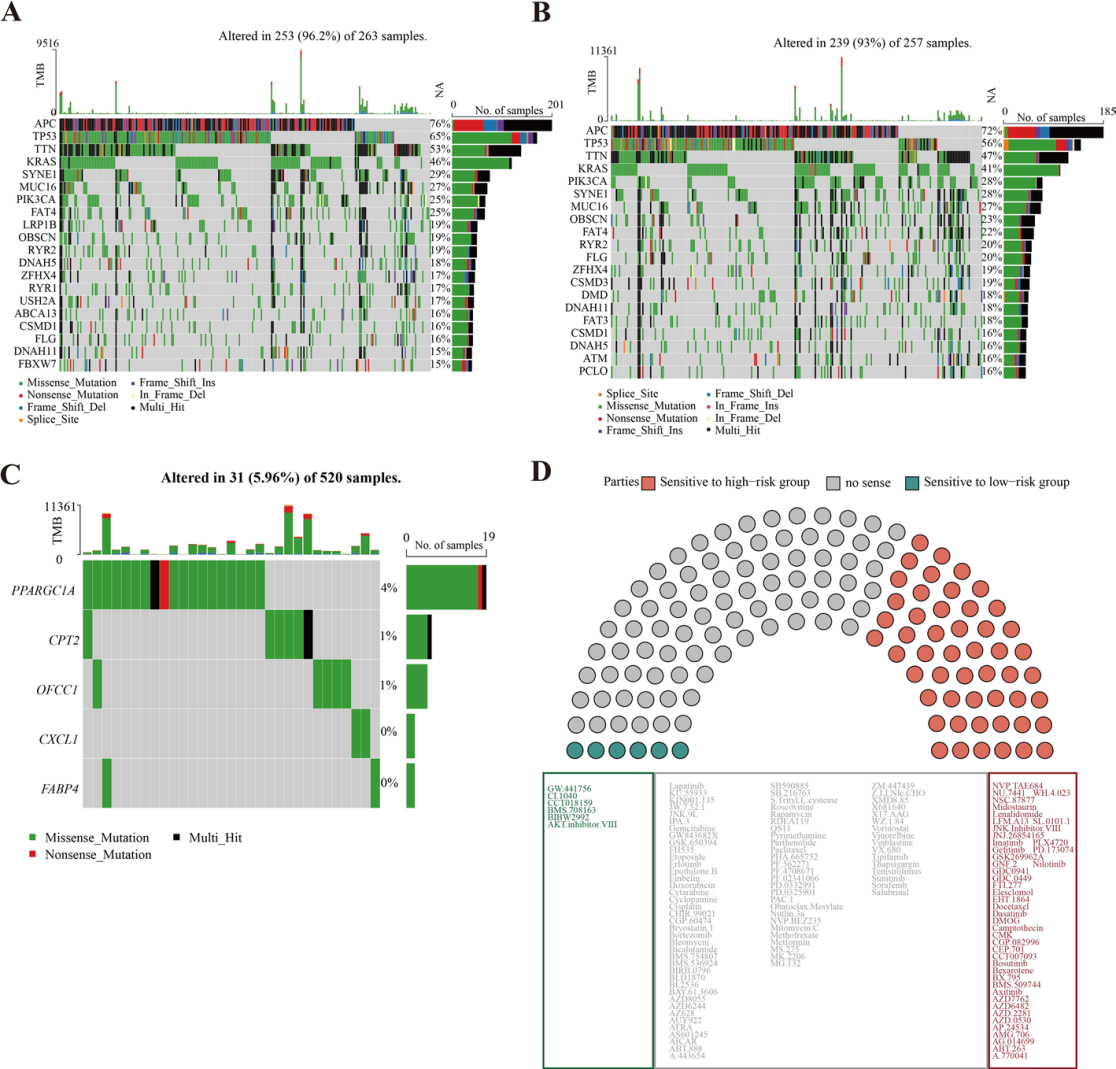
Genomic mutations and drug sensitivity testing. **A**, **B** Waterfall plots of the top 20 mutated genes in the high-risk (A: APC 76%, TP53 65%) and low-risk (B: APC 72%, TP53 56%) groups. **C** Missense mutations predominant in prognostic LRGs. **D** Drug sensitivity analysis showing higher sensitivity to midostaurin and lenalidomide (*p* < 0.05) in high-risk patients and to AKT inhibitors in low-risk patients. (Mutation data from the TCGA-CRC cohort; drug IC_50_ obtained via the GDSC database)

After the analysis of transcriptional variations (reported above in Sect. 3.5), we proceeded to explore whether there was any evidence indicating disparities at the genomic level between the two risk groups. The mutant gene waterfall plots highlighted the top 20 genes with the highest mutation frequencies. In the high-risk group, the top five mutated genes were *APC*, *TP53*, *TTN*, *KRAS*, and *SYN1*, with *APC* showing the highest mutation rate of 76%, followed by *TP53* at 65% (Fig. 6A). In the low-risk group, the top five mutated genes comprised *APC*, *TP53*, *TTN*, *KRAS*, and *PIK3CA*, with *APC* and *TP53* exhibiting mutation rates of 72% and 56%, respectively (Fig. 6B). Fascinatingly, *APC*, *TP53*, *TTN*, and *KRAS* consistently occupied the top four positions in both groups, reflecting pivotal roles in CRC modulation [31], and the prognostic LRGs predominantly underwent missense mutation (Fig. 6C). The observed genomic mutations revealed the crucial roles played by genes like *APC*, *TP53*, *TTN*, and *KRAS* in CRC progression and the predominance of missense mutations in prognostic LRGs.

With the aim of enhancing prognosis prediction for patients with CRC, drug sensitivity testing was employed to predict the responsiveness of each patient to a spectrum of drugs based on their gene expression profiles. Overall, the results revealed significant discrepancies in the IC_50_ values for 57 drugs. Low-risk patients demonstrated greater sensitivity toward GW.441,756, CI.1040, CCT018159, BMS.708,163, BIBW2992, and AKT.inhibitor.VIII compared to the high-risk patients (*p* < 0.05). Nevertheless, the latter exhibited lower IC_50_ values for numerous chemotherapeutic agents, such as NVP.TAE684, midostaurin, lenalidomide, elesclomol, and dasatinib (*p* < 0.05), implying that they tended to display reduced resistance to chemotherapy compared to low-risk patients (Fig. 6D). In conclusion, the results of drug sensitivity testing suggested that the drugs examined have the potential to improve CRC treatment by targeting lipotoxicity.

### Target miRNA prediction and construction of TF networks

**Fig. 7:**
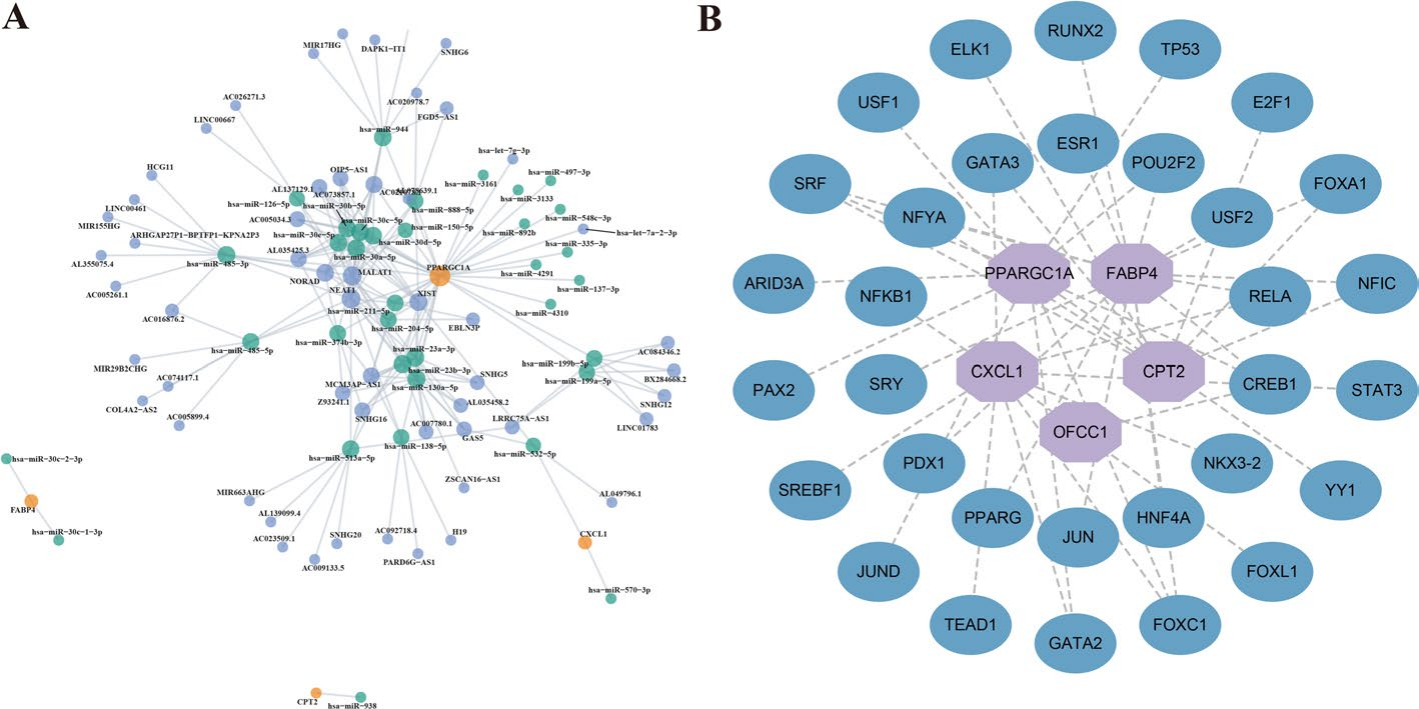
Regulatory networks of prognostic LRGs. **A** lncRNA–miRNA–mRNA network (e.g., *NEAT1/hsa-miR-30c-5p/PPARGC1A*). **B** TF–mRNA network showing key transcription factors (e.g., NFKB1, PPARG) regulating prognostic LRGs. (Data from StarBase and NetworkAnalyst)

It is well known that miRNAs can silence genes and downregulate gene expression by binding to mRNAs. LncRNAs, which are considered upstream molecules, can influence the function of miRNAs by binding to miRNA response elements and further upregulating gene expression [32]. In this study, a total of 157 and 126 predicted miRNAs were identified for prognostic LRGs from the StarBase database and miRDB database, respectively. A total of 22 target miRNAs (including hsa-miR-30c-5p, hsa-miR-30a-5p, hsa-miR-150-5p, hsa-miR-211-5p, and hsa-miR-374b-5p) were retrieved, and their associations with lncRNAs were predicted. The results yielded 53 target lncRNAs (including LINC00667, NEAT1, MALAT1, AC026271.3, SNHG16, GAS5, AC007780.1, SNHG5, XIST, ZSCAN16-AS1, and Z93241.1) for miRNAs predicted for prognostic LRGs from the StarBase database (Fig. 7A). Upstream regulation of prognostic LRGs was explored by predicting relevant TFs using the NetworkAnalyst database, and an mRNA–TF regulatory network was established. Specifically, seven TFs were predicted for *CPT2*, 10 for *CXCL1*, 15 for *FABP4*, 1 for *OFCC1*, and 11 for *PPARGC1A*. Among them, several TFs with strong associations were identified, namely *GATA3*, *NFKB1*, *SRY*, *PPARG*, *JUN*, *HNF4A*, *CREB1*, *RELA*, and *USF2* (Fig. 7B). This network exploration allowed thorough elucidation of the regulatory mechanisms of lipotoxicity in CRC.

### Experimental validation of prognostic LRGs

**Fig. 8:**
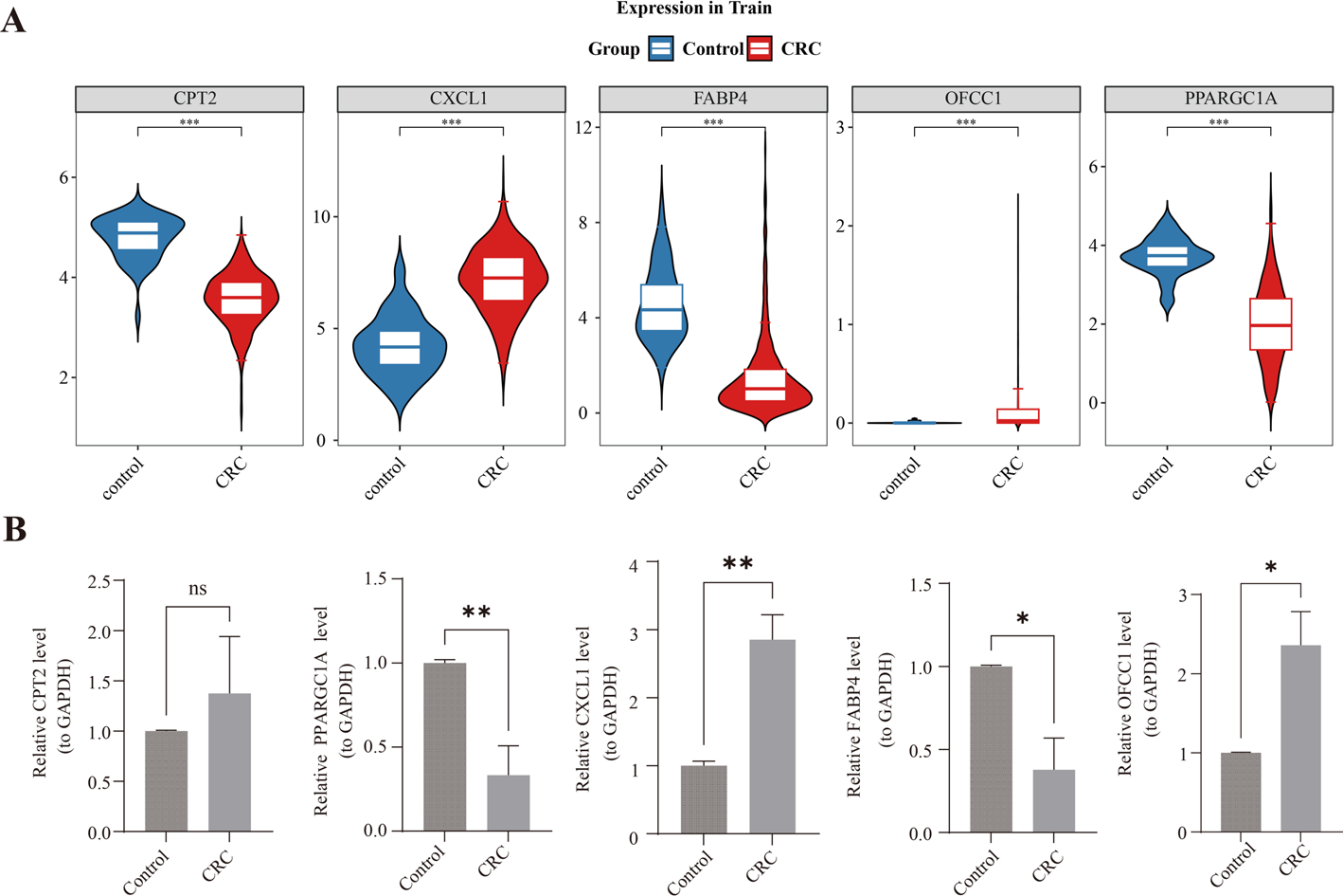
Experimental validation of prognostic LRGs. **A** Violin plots showing the expression levels of LRGs from the TCGA database (***p < 0.001). s**B** RT-qPCR validation in clinical samples. (ns = not significant, *p < 0.05, **p < 0.01). GAPDH was used as internal control; error bars: SD

Comparative analysis of the CRC and control groups in the TCGA-CRC dataset to confirm the differences in prognostic LRG expression (i.e., *PPARGC1A*, *CPT2*, *CXCL1*, *FABP4*, and *OFCC1*) showed significantly elevated expression levels for *CXCL1* and *OFCC1* (*p* < 0.001) and significantly reduced levels for *FABP4*, *PPARGC1A*, and *CPT2* (*p* < 0.001) in patients with CRC (Fig. 8A). The results of RT-qPCR were similar to those obtained for TCGA-CRC dataset analysis (Fig. 8B), demonstrating that these prognostic LRGs might play crucial roles in the development and progression of CRC.

## Discussion

In this study, we employed a series of bioinformatics approaches to evaluate the expression levels and prognostic value of LRGs in patients with CRC, identifying five potential prognostic LRGs (*PPARGC1A*, *CPT2*, *CXCL1*, *FABP4*, and *OFCC1*)[33]. The underlying regulatory mechanisms of these prognostic genes were further investigated via functional enrichment analysis, immune infiltration analysis, and other methods, providing novel insights for the prognosis and immunotherapy of patients with CRC [34]. Furthermore, our findings were consistent between training set analysis and RT-qPCR validation, demonstrating significantly elevated expression levels for *CXCL1* and *OFCC1* and significantly reduced levels for *FABP4*, *PPARGC1A*, and *CPT2* in patients with CRC [35]. These results suggest that prognostic LRGs represent potential therapeutic targets for CRC treatment.

Downregulation of PPARGC1A, a master regulator of mitochondrial biogenesis and energy metabolism, directly impairs the capacity for fatty acid oxidation in CRC cells. This leads to abnormal intracellular lipid accumulation and triggers lipotoxic stress [36], which may further drive tumor proliferation by suppressing AMPK and activating the mTOR signaling pathway [37, 38]. In line with this, overexpression of PPARGC1A in CRC cells induces substantial accumulation of mitochondrial reactive oxygen species and ultimately promotes apoptosis [39], revealing the tumor-suppressive potential of this regulator. Moreover, the functional status of PPARGC1A profoundly shapes the tumor immune microenvironment. Research has shown that activators of PGC-1α (the protein encoded by PPARGC1A) enhance the function of tumor-infiltrating cytotoxic T cells by promoting fatty acid oxidation, thereby improving the efficacy of PD-1 blockade in lung cancer models [40]. Notably, MIER2 can suppress the expression of PGC-1α by recruiting HDAC1 and mediating the deacetylation of p53, ultimately leading to lipid accumulation and contributing to drug resistance in renal cell carcinoma [41].

Carnitine palmitoyltransferase 2 (CPT2) facilitates mitochondrial fatty acid transport. Recent studies have validated its role as a prognostic biomarker in CRC, where its decreased expression correlates with adverse outcomes. Mechanistically, CPT2 downregulation promotes tumor cell proliferation while suppressing apoptosis through p53 pathway inactivation, indicating the tumor-suppressive function of this enzyme in CRC pathogenesis. Additionally, studies have shown that inhibition of CPT2 can enhance selective autophagy and proliferation in CRC cells via a GPAT4-dependent glycerophospholipid biosynthetic pathway [42]. Similarly, in hepatocellular carcinoma models, knockdown of CPT2 has been shown to significantly increase the tumorigenicity and metastatic potential of cancer cells and induce chemoresistance to cisplatin. Mechanistically, low CPT2 expression upregulates stearoyl-CoA desaturase-1—a key enzyme involved in monounsaturated fatty acid synthesis—thereby promoting lipogenesis in cancer cells [43]. These findings indicate that CPT2 plays a pivotal role in tumor progression and chemotherapeutic response by modulating lipid metabolism and apoptotic pathways, and its expression level is closely associated with patient prognosis and therapeutic sensitivity.

C–X–C motif ligand 1 (CXCL1) is a chemokine that exhibits oncogenic properties in KRAS-mutated CRC. Mitochondrial glutamate carrier SLC25A22 drives CXCL1 transcription via the SLC25A22–asparagine–SRC/ETS2 signaling axis [32]. Secreted CXCL1 then recruits myeloid-derived suppressor cells (MDSCs) through CXCR2 binding, establishing an immunosuppressive microenvironment that accelerates tumor progression [44]. Tumor-secreted VEGFA can stimulate tumor-associated macrophages to produce CXCL1, which subsequently recruits CXCR2-positive MDSCs to form a pre-metastatic niche in the liver [44, 45]. Notably, a potential link exists between dysregulated lipid metabolism and CXCL1-mediated immune suppression. Studies have shown that elevated FFAs upregulate hepatic CXCL1 expression and promote neutrophil infiltration, thereby inducing inflammatory damage [46]. In our study, TF network analysis revealed a potential regulatory relationship between SREBF1 and CXCL1. This suggests that in CRC, lipotoxic stress may similarly activate the CXCL1 signaling axis, thereby reshaping the immune microenvironment.

Fatty acid–binding protein 4 (FABP4) serves as a key mediator for remodeling the CRC microenvironment, exerting multiple pro‑tumor functions through the regulation of lipid metabolism. Its overexpression enhances fatty acid transport, activates the AKT signaling pathway, and drives the epithelial–mesenchymal transition, thereby directly promoting tumor cell migration and invasion [47]. At the tumor microenvironment level, aberrant *FABP4* expression modulates the distribution of lipids and their metabolites, influencing oxidative stress, angiogenesis, and immune cell function [48]. Notably, *FABP4* shows a significant correlation with the infiltration of NK and NKT cells, suggesting its involvement in immunometabolic regulation. Recent studies have highlighted the critical role of metabolic reprogramming in shaping the immunosuppressive tumor microenvironment [49]. Specifically, the TF MEF2C has been shown to regulate NK cell antiviral function by modulating SREBP‑mediated lipid metabolism [50], whereas dysregulated cholesterol metabolism impairs NKT cell activity and weakens antitumor immune surveillance [51]. In our study, we further identified a potential regulatory relationship between the TF JUN and FABP4, indicating that its upstream regulatory network may participate in the crosstalk between lipid metabolism and the immune microenvironment. Therefore, we propose that FABP4 likely drives CRC progression through a dual mechanism: on one hand, it directly activates the AKT–EMT axis in tumor cells; on the other hand, it remodels lipid metabolism within the tumor microenvironment, affecting the metabolic fitness and function of NK/NKT cells and ultimately promoting immune escape.

*OFCC1* is a non-coding RNA gene whose full name is Orofacial Cleft 1 Candidate 1. Research suggests that the retained intergenic domain within the OFCC1 locus is likely to be an oncogenic mutation [52]. The role of this gene in tumor progression remains unclear, although elevated expression levels may indicate advanced disease stages. Our study is the first to demonstrate that OFCC1 has a role in CRC, providing novel insights into CRC prognosis.

Our GSEA identified significant enrichment of three key signaling pathways across risk groups: PPAR signaling [53–55], adipocytokine signaling [56–58], and AMPK signaling [59–61]. These pathways collectively regulate lipid metabolism, energy homeostasis, and inflammatory responses—processes central to lipotoxicity-driven carcinogenesis [53]. As a master regulator of fatty acid oxidation, storage, and glucose metabolism, the PPAR signaling pathway modulates cellular differentiation and inflammation [54]. In CRC, dysregulation of this pathway promotes tumor progression by altering lipid catabolism and creating a permissive microenvironment for cancer cell proliferation [55]. Our findings align with previous studies linking PPAR pathway activation to CRC aggressiveness and metabolic reprogramming.

Adipocytokines (e.g., leptin and adiponectin) bridge adipose tissue dysfunction with systemic inflammation and insulin resistance. In CRC, aberrant adipocytokine signaling exacerbates tumor growth by activating pro-inflammatory cascades (e.g., JAK/STAT and NF-κB) and suppressing antitumor immunity [56]. The observed enrichment in this pathway underscores the mechanistic interplay between adiposity-related lipotoxicity and CRC pathogenesis, corroborating prior evidence of its prognostic relevance [57]. AMPK acts as a cellular energy sensor that regulates anabolic/catabolic balance. Its disruption in CRC impairs lipid homeostasis, promoting ectopic lipid accumulation and mitochondrial dysfunction [59]. AMPK inactivation further accelerates tumor progression by enabling unchecked mTOR-driven proliferation and evasion of metabolic stress [61]. Our results support its role as a critical component of a tumor-suppressive axis that becomes compromised in lipotoxicity-associated CRC.

The convergence of the above-mentioned pathways in our analysis highlights a coordinated metabolic immune dysregulation in high-risk patients with CRC. The PPAR and adipocytokine pathways drive lipid overload and inflammation, while AMPK failure exacerbates energy imbalance, collectively fueling tumor growth and immune evasion. The observed enrichment in these three pathways substantiates the biological plausibility of lipotoxicity as a therapeutic target and validates the ability of our risk model to stratify patients with distinct metabolic vulnerabilities.

Our immune infiltration analysis revealed significant disparities in tumor microenvironment composition between risk groups. High-risk patients with CRC exhibited elevated infiltration of CD56dim NK cells, NK cells, NK T cells, and T follicular helper cells, whereas low-risk patients showed enrichment in type 17 T helper cells, neutrophils, and activated CD4 T cells (*p* < 0.05; Fig. 5A, B). Strikingly, FABP4 demonstrated the strongest positive correlation with total NK cells (cor = 0.58, *p* < 0.05) and NK T cells (cor = 0.53, *p* < 0.05; Fig. 5C).

NK cells, as critical effectors of antitumor immunity, eliminate malignant cells via cytotoxic granule release and death receptor signaling [62]. In CRC, impaired NK cell activity correlates with advanced disease stages and metastasis. The synergy between FABP4 and NK cells observed in this study suggests that lipotoxicity may remodel immune surveillance—a finding supported by studies linking NK dysfunction to poor CRC outcomes [63]. Activated CD4 T cells coordinate adaptive immunity by modulating T helper responses. Their depletion in high-risk patients (*p* < 0.05; Fig. 5B) indicates compromised immune priming, potentially enabling tumor immune evasion—in line with reports of CD4 T cell deficiency in progressive CRC.

We further identified 16 differentially expressed immune checkpoints between risk groups (*p* < 0.05; Fig. 5D). Notably, CD48 (a regulator of NK/T cell activation) showed the strongest positive correlation with FABP4 (cor = 0.55, *p* < 0.05; Fig. 5E), which aligns with evidence that CD48 blockade enhances antitumor immunity in CRC models. These findings collectively demonstrate that prognostic LRGs—particularly FABP4—orchestrate immune evasion in CRC through three interconnected mechanisms: recruitment of immunosuppressive NK subsets, depletion of antitumor CD4⁺ T cells, and upregulation of checkpoint networks (e.g., CD48–LAG3 axis). Understanding these pathways contributes to advancing personalized immunotherapy through (1) targeted FABP4 inhibition to restore NK cytotoxicity, (2) combinatorial regimens merging checkpoint blockers (e.g., anti-LAG3) with modulators of lipid metabolism, and (3) precision stratification using LRG-immune signatures.

Our drug sensitivity analysis identified midostaurin and lenalidomide as clinically actionable agents demonstrating divergent efficacy across CRC risk groups (Fig. 6D), highlighting their therapeutic potential for lipotoxicity-associated CRC. Midostaurin, a multi-kinase inhibitor primarily indicated for acute myeloid leukemia and systemic mastocytosis, exhibits repositioning potential for CRC through dual mechanisms [64]. Its polypharmacological profile targets oncogenic kinases (e.g., FLT3, PKCβ, and VEGFR) that are hyperactivated in CRC signaling cascades. Crucially, midostaurin’s anti-angiogenic activity via VEGFR suppression disrupts tumor neovascularization, a process essential for CRC growth and metastasis, thereby restricting nutrient delivery and metastatic dissemination [65]. This kinase-targeted approach aligns with precision oncology paradigms for metabolic-based intervention.

Lenalidomide exerts multimodal activity against CRC beyond its hematological indications, namely (1) direct antiproliferative effects on malignant cells, (2) potent angiogenesis inhibition through VEGF pathway suppression, and (3) immunological reprogramming via enhanced T cell/NK cell cytotoxicity [66]. This tripartite mechanism simultaneously targets tumor proliferation, microenvironmental support, and immune evasion, positioning lenalidomide as a strategic candidate for combinatorial regimens in lipotoxicity-associated CRC, particularly given its established safety profile in clinical practice.

In summary, our study established a prognostic risk model for CRC based on LRGs and demonstrated its independent predictive value for patient outcomes. In parallel, recent studies have applied molecular subtyping using immune-related gene signatures to stratify patients with CRC into distinct prognostic subtypes, identifying key genes such as SPP1 as biomarkers for predicting immune status and prognosis [67]. These findings further validate the clinical utility of multi-gene molecular profiling for accurate prognostic assessment of CRC. Moving forward, integration of both the “metabolic” and “immune” dimensions may offer a novel molecular framework for developing more accurate and individualized therapeutic strategies.

## Conclusions

Based on bioinformatic analysis and preliminary RT-qPCR validation, we developed a prognostic model for CRC comprising five LRGs (*PPARGC1A*, *CPT2*, *CXCL1*, *FABP4*, and *OFCC1*). By integrating risk stratification, pathway enrichment analysis, tumor microenvironment analysis, and drug sensitivity assessment, our model provides a novel framework for prognostic evaluation and therapeutic decision‑making in CRC. However, several limitations should be acknowledged. The study is inherently exploratory and hypothesis‑generating in nature. Although RT‑qPCR confirmed differential expression of the core genes, the sample size was limited, and functional experiments are still required to establish causal roles and elucidate specific molecular mechanisms in CRC development. Furthermore, the findings of this type of research depend on the quality of public databases and the robustness of algorithmic models, which may be influenced by data noise, model complexity, and statistical biases. Future investigations should prioritize functional assays to define how genes such as *OFCC1* and *FABP4* regulate lipid metabolism, proliferation, and invasion in CRC cells. Mechanistic studies are also needed to investigate how key genes modulate immune cell infiltration and function within the tumor microenvironment. Moreover, multicenter prospective clinical validation is essential. Through blinded study designs, the prognostic stratification ability of our risk model should be evaluated in independent cohorts, and its potential to identify patient subgroups that may benefit from specific therapies should be explored, thereby laying the groundwork for future precision‑medicine clinical trials.

**Table 1:** The primer sequences

Primer	Sequence
CXCL1-F	AGGCAGGGGAATGTATGTGC
CXCL1-R	GCCCCTTTGTTCTAAGCCAGA
OFCC1-F	GAGAGCAGACAAGGAGAACCC
OFCC1-R	TTGGAACTCCTCAAGGTGAGC
FABP4-F	AATGCGTCATGAAAGGCGTC
FABP4-R	ATGCGAACTTCAGTCCAGGT
PPARGC1A-F	CCCCATGGATGAAGGGTACTT
PPARGC1A-R	GGGGAGGTCTCATCCATTGC
CPT2-F	GTAGCACTGCCGCATTCAAG
CPT2-R	GGCCATGGTACTTGGAGCAC
GAPDH-F	ATGGGCAGCCGTTAGGAAAG
GAPDH-R	AGGAAAAGCATCACCCGGAG

## Data Availability

The datasets generated and/or analyzed during the current study are available in public repositories. The Cancer Genome Atlas (TCGA) Colon Adenocarcinoma (COAD) and Rectum Adenocarcinoma (READ) datasets (collectively termed TCGA-CRC) were downloaded from the UCSC Xena database (https://xena.ucsc.edu/). The validation dataset GSE87211 was obtained from the Gene Expression Omnibus (GEO) database (https://www.ncbi.nlm.nih.gov/geo/query/acc.cgi? acc=GSE87211). The list of 467 lipotoxicity-related genes (LRGs) was sourced from the GeneCards database (https://www.genecards.org/). Drug sensitivity data were derived from the Genomics of Drug Sensitivity in Cancer (GDSC) database (https://www.cancerrxgene.org/). Other datasets used and/or analyzed are available from the corresponding author upon reasonable request.
